# Mature cystic teratoma of the fallopian tube associated with incomplete uterine mediastinum: A case report and literature review

**DOI:** 10.3892/ol.2013.1355

**Published:** 2013-05-20

**Authors:** SHUYI LI, XIAOLING FANG, JIANLIN CHEN, XIAOMENG XIA

**Affiliations:** Department of Obstetrics and Gynaecology, The Second Xiangya Hospital, Central South University, Changsha, Hunan 410000, P.R. China

**Keywords:** teratoma, fallopian tube, infertility, incomplete uterine mediastinum

## Abstract

Neoplasms of the fallopian tube are extremely uncommon. In addition, the incidence of a mature teratoma of the fallopian tube with incomplete uterine mediastinum is extremely low. In the current case report, we present a case of mature cystic teratoma of the fallopian tube with incomplete uterine mediastinum in a 23-year-old female. This mass was noted on computed tomography (CT) scan and sonographic examination. The patient underwent left salpingectomy and uterine septum incision during surgery. One year later, the individual became pregnant.

## Introduction

Neoplasms of the fallopian tube are the least common tumors of the female reproductive system. To date, mature teratoma of the fallopian tube associated with incomplete uterine mediastinum has not been reported and ∼60 cases of mature teratoma of the fallopian tube have been reported worldwide. None of the reported cases were diagnosed preoperatively. The pathogenesis of teratoma of the fallopian tube remains poorly understood. Tumors are accidentally discovered and confirmed by pelvic surgery, as a result of diagnosis of uterine fibroids, ovarian cysts and cesarean sections. It has been hypothesized that teratomas originate from the germ cells, as teratomas are found most frequently in the gonads. The karyotype of all benign teratomas is 46,XX. Surgery is used to treat teratomas and the prognosis of this disease is generally good (1–5). In the present case report, the patient was presented to The Second Xiangya Hospital of Central South University (Changsha, China) with fertility issues. The postoperative diagnosis was incomplete uterine mediastinum and mature cystic teratoma of the fallopian tube. One year after surgery, the patient became pregnant and gave birth to a healthy baby. Written informed consent was obtained from the patient.

## Case report

### Patient presentation

**Figure 1.**
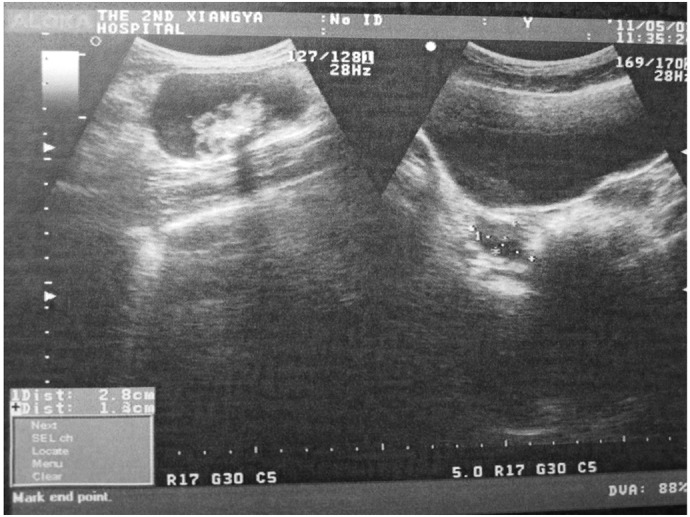
Sonographic examination of the pelvis revealed a cystic mass of ∼8×4 cm in the right hypogastic region.

**Figure 2.**
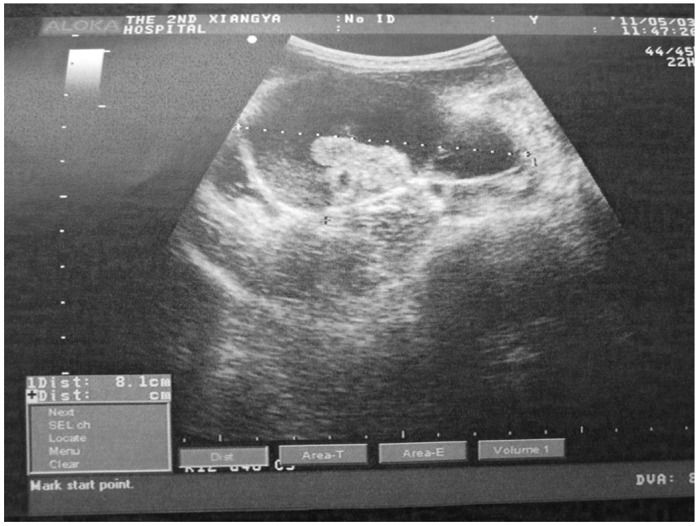
Sonographic examination of the pelvis revealed that the mass contained foci of calcification of ∼3×2 cm.

**Figure 3.**
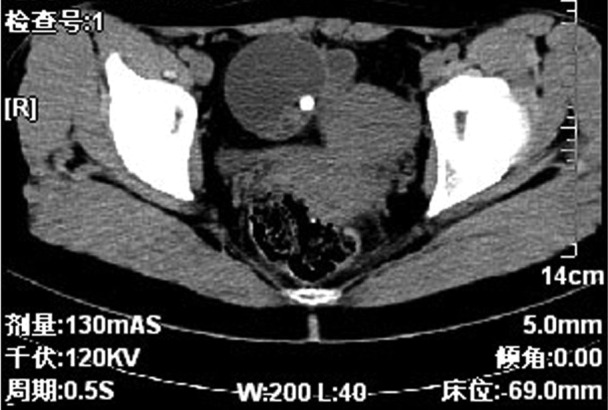
Pelvic computed tomography (CT) scan revealed a 6×4.5 cm cystic mass to the left of pelvis.

A 23-year-old female (*gravida* 0, *para* 0) who had been married for two years was admitted to The Second Xiangya Hospital of Central South University (Changsha, China) with fertility issues. The patient reported intermittent lower abdominal pain for 8 months. The individual’s menstrual cycle was regular. Pelvic examination revealed a stationary cystic mass of ∼8×3 cm in the left adnexal region; the right adnexa and the uterus were normal. The patient’s family history was negative for any hereditary diseases. Serum concentrations of C12 were within the normal limits. Ultrasound examination revealed that the endometrium was unclear, and a cystic mass of ∼8×4 cm to the right of hypogastic region was observed. The mass contained foci of calcification of ∼3×2 cm (Figs. 1 and 2). A pelvic computed tomography (CT) scan revealed a 6×4.5 cm cystic mass in the left pelvis, which was suspected to represent a cystic teratoma of the left ovary (Fig. 3). The density of the uterine body was irregular as shown by enhanced B sonography, which did not eliminate the presence of uterine disease.

### Surgical procedures

**Figure 4.**
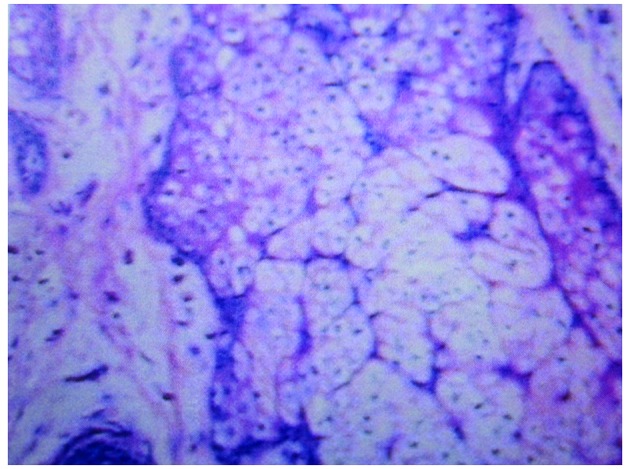
Microscopic observations of the mature teratoma demonstrate a cyst lined entirely by well-differentiated keratin-producing squamous epithelium (magnification, ×100).

**Figure 5.**
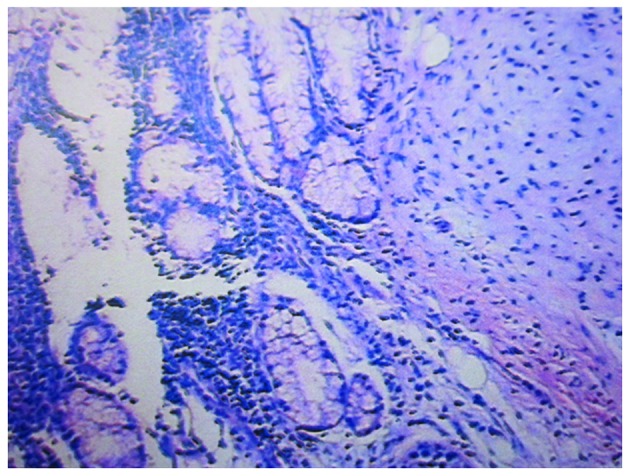
Microscopic mature teratoma observations (magnification, ×100).

The patient underwent surgery with a diagnosis of cystic teratoma of the left ovary, incomplete uterine mediastinum and infertility. At the time of surgery, incomplete mediastinum (∼2 mm) was observed at the bottom of the uterus. The left fallopian tube appeared to be distended by ∼8×3 cm, and contained cream-colored sebaceous material and hair. The left ovary and the right adnexa were normal. The patient subsequently underwent left salpingectomy and uterine septum incision. The post-operative recovesry was uneventful. The ampullary portion of the left fallopian tube was histologically diagnosed as mature cystic teratoma (Figs. 4 and 5).

## Discussion

Tubal teratoma may be associated with other neoplasms (6,7), struma ovarii, ectopic pregnancy (8,9), uterine leiomyomatosis (10) and endometrial adenocarcinoma (11) (Table I). The present case study is the first in the literature to report a mature cystic teratoma of the fallopian tube associated with incomplete uterine mediastinum (11). In addition, the teratoma in the present case was the largest of all reported cases of mature cystic teratomas of the fallopian tube. The diversity of teratoma behavior is likely to reflect the varied biological potential of different stem cells, including germ and pluripotent embryonic cells. Benign teratoma of the fallopian tube is composed of tissues of ectodermal, mesodermal and endodermal origin in any combination (1). Teratomas are initially of mesodermal origin with abundant mesenchymal stroma; however, it eventually develops endodermal and ectodermal derivatives with airway-lining enterocytes, thyroid, brain and skin appendages (12). The majority of cases of benign teratoma of the fallopian tube occur in patients in their 40s and are cystic and exhibit significant variations in size. The majority of benign teratomas of the fallopian tube are unilateral and are common in 1/3 of the fallopian tube or the outer edge of the fallopian tube. The tumors tend to be small and contain sebum-like material with hairs. Fallopian tube teratomas have been associated with reduced parity, menstrual irregularity, leukorrhea, post-menopausal bleeding and abdominal pain. Procedures, including analysis of serum C12 levels, pelvic CT scan, ultrasound examination (13) and hysterosalpingography (12), are useful for diagnosis. The prognosis of the disease is good. In the present case, the patient was 23 years-old; however, the teratoma was larger compared with other studies (14–19). The majority of tubal teratomas are benign. In the current case, the patient suffered from incomplete uterine mediastinum, which was due to paramesonephric duct convergence insufficiency. Intermittent lower abdominal pain has been reported in other cases, consistent with the current case; however, the patient was originally admitted to hospital due to infertility. One year following the surgery, the individual became pregnant.

In conclusion, benign teratomas commonly occur in the ovaries, but are rarely found in the fallopian tubes. Although the present case was a cystic teratoma, immature tissues were not identified by microscopic examination of the specimens by several pathologists. Therefore, a diagnosis of a benign mature cystic teratoma originating in the fallopian tube was reported for this patient.

## Figures and Tables

**Table I.** Tubal teratomas in the literature.

First author (ref.)	Complication	Age, years	Gravidity^a^ and parity	Side	Size, cm	Other
Baginski	Ectopic pregnancy	33	G2P2	L	Egg	Increased hCG
Roberts	Ectopic pregnancy	29	G0P0	L	4×2	Increased hCG
Neumann	Ectopic pregnancy	36	G0P0	L	3.4×1	Increased hCG
Zelinger	Ectopic pregnancy	30	G2P2	L	1×1.5	Increased hCG
Massouda	Ectopic pregnancy	24	G0P0	R	2.5×1	Increased hCG
Kutteh	Ectopic pregnancy	25	G3P3	L	2×2	Increased hCG
Chao	Uterine leiomyomatosis	40	G6P2	L	4.0×2.2×1.2	Normal C12
Hoda	Struma salpingis	44		L	0.2	A 7.0-cm mucinous cystadenoma of the contralateral ovary
Roncati	Endometrial adenocarcinoma	67	G4P4	L	0.5×0.6	
PC	Incomplete uterine Mediastinum	23	G0P0	L	8×3	Normal C12

a Gravidity does not include the ectopic gestation at the time of presentation. PC, present case; G, gravidity; P, parity; L, left; R, right.
